# Metalloprotease Dependent Release of Placenta Derived Fractalkine

**DOI:** 10.1155/2014/839290

**Published:** 2014-03-13

**Authors:** Monika Siwetz, Astrid Blaschitz, Julia Kremshofer, Jelena Bilic, Gernot Desoye, Berthold Huppertz, Martin Gauster

**Affiliations:** ^1^Institute of Cell Biology, Histology and Embryology, Medical University Graz, Harrachgasse 21/VII, 8010 Graz, Austria; ^2^Department of Obstetrics and Gynaecology, Medical University Graz, Harrachgasse 21/VII, 8010 Graz, Austria

## Abstract

The chemokine fractalkine is considered as unique since it exists both as membrane-bound adhesion molecule and as shed soluble chemoattractant. Here the hypothesis was tested whether placental fractalkine can be shed and released into the maternal circulation. Immunohistochemical staining of human first trimester and term placenta sections localized fractalkine at the apical microvillous plasma membrane of the syncytiotrophoblast. Gene expression analysis revealed abundant upregulation in placental fractalkine at term, compared to first trimester. Fractalkine expression and release were detected in the trophoblast cell line BeWo, in primary term trophoblasts and placental explants. Incubation of BeWo cells and placental explants with metalloprotease inhibitor Batimastat inhibited the release of soluble fractalkine and at the same time increased the membrane-bound form. These results demonstrate that human placenta is a source for fractalkine, which is expressed in the syncytiotrophoblast and can be released into the maternal circulation by constitutive metalloprotease dependent shedding. Increased expression and release of placental fractalkine may contribute to low grade systemic inflammatory responses in third trimester of normal pregnancy. Aberrant placental metalloprotease activity may not only affect the release of placenta derived fractalkine but may at the same time affect the abundance of the membrane-bound form of the chemokine.

## 1. Introduction

During human gestation the placenta as a temporal villous organ fulfills a wide spread panel of pregnancy maintaining functions, including exchange of gases and metabolites, regulation of water balance, and secretion of endocrine factors. The vast majority of placenta derived endocrine factors are synthesized in the syncytiotrophoblast, which—as a unique epithelium-like layer without lateral cell borders—covers all placental villous trees as well as parts of the inner surfaces of chorionic and basal plates. Thus, the syncytiotrophoblast lines the intervillous space and hence is exposed to maternal blood [1]. Beside analogues of virtually all known classical hypothalamic and pituitary hormones, the human syncytiotrophoblast also synthesizes steroid hormones, monoamines, adrenal-like peptides, cytokines, and chemokines [2, 3].

Chemokines are classified into four subfamilies according to the number and spacing of the first two cysteine residues in a conserved cystein structural motif [4]. These four subclasses are referred to as C, CC, CXC, and CX_3_C, where C is a cysteine and X any amino-acid residue. The CX_3_C subclass was discovered in the late 1990s and contains only one member, termed fractalkine, or CX3CL1 [5]. Fractalkine is synthesized as a 373 amino-acid transmembrane molecule, comprising an extracellular N-terminal domain, a mucin-like stalk, a transmembrane *α*-helix, and a short cytoplasmic tail [6, 7]. The extracellular domains, representing the chemokine domain and the mucin-like stalk, can be shed by metalloproteases into a soluble isoform [8–10]. Thus, fractalkine exists as both, a membrane-bound and a soluble form—a situation considered as unique amongst the group of chemokines. While the soluble form has chemoattractive activity for monocytes, natural killer cells, and T-cells, the membrane-bound form promotes flow resistant adhesion of leukocytes to endothelial cells via its corresponding G protein-coupled, 7-transmembrane receptor CX3CR1 [11]. Based on that, fractalkine may be considered as inflammatory chemokine expressed in activated endothelial and epithelial cells, as well as in dendritic cells, lymphocytes, osteoblasts, neurons, and microglial cells [12–14]. According to tissue distribution analysis fractalkine mRNA is most abundantly expressed in brain, heart, kidney, lung, and pancreas but can also be detected in human placenta [5].

However, current knowledge on placenta derived fractalkine and its implications on pregnancy is limited and based on a small number of studies. Placental fractalkine expression was initially demonstrated in villous trophoblast and the amniotic epithelium, which was suggested as resource for substantial release of soluble fractalkine into amniotic fluid of human second and third trimester pregnancies [15]. Studies by Hannan et al. showed fractalkine expression by semiquantitative RT-PCR in primary endometrial epithelial cells and the trophoblast cell lines JEG-3, AC1M-32, and AC1M-88 [16]. Migration and adhesion studies by the same group suggested fractalkine to be involved in embryo implantation processes [17]. Recently, increased placental fractalkine expression was suggested to contribute to increased microvessel density in placental tissue from pregnancies complicated by diabetes mellitus [18].

In the light of the broad panel of factors released from human placenta it could be assumed that fractalkine is one of them. However, experimental evidence for placental fractalkine release has not been provided so far. Thus, we aimed to analyse the spatiotemporal expression of placental fractalkine and tested the hypothesis whether it can be shed and released into the intervillous space, that is, the maternal circulation.

## 2. Methods

### 2.1. Human Placenta Tissue Samples

The study was approved by the ethical committee of the Medical University of Graz and informed consent was obtained from the women. First trimester placentas (mean gestational week: 9.4 ± 1.7) were obtained from women (mean maternal age: 28.1 ± 6.2 years; mean body mass index: 24.2 ± 5.0) undergoing pregnancy terminations for psychosocial reasons. Term placentas were obtained after delivery (mean gestational age: 39.4 ± 0.9 weeks) from healthy women (mean maternal age: 34.8 ± 3.7 years; mean body mass index: 23.4 ± 4.4) with singleton pregnancies and no clinical evidence of infection. Pregnancies complicated by hypertension, preeclampsia, metabolic disease, steroid treatment, AIDS, alcohol abuse, and/or drug abuse were excluded.

### 2.2. Immunohistochemistry

Formalin fixed and paraffin-embedded (FFPE) tissue sections (5 *μ*m) from ten first trimester and ten term placentas were mounted on Superfrost Plus slides (Menzel/Thermo Fisher Scientific). After standard deparaffination, tissue sections were subjected to antigen retrieval by boiling slides in Epitope Retrieval Solution pH 9.0 (Novocostra, Leica) for 7 min at 120°C in a decloaking chamber (Biocare Medical). Sections were immunostained using a staining robot (Autostainer 360, Thermo Fisher Scientific) and the UltraVision Large Volume Detection System HRP Polymer Kit (Thermo Fisher Scientific) according to manufacturer's instruction. In brief, hydrogen peroxidase block was used for 10 min to block endogenous peroxidase. Washing steps with TBS including 0.05% Tween 20 (TBS/T; Merck) were followed by background blocking with Ultra V Block for 5 min. Monoclonal anti-human CX3CL1/fractalkine antibody (clone 81513, R&D Systems) was diluted 1 : 1000 (0.5 *μ*g/mL working concentration) in Antibody Diluent (Dako) and incubated on slides for 30 min at RT. After TBS/T washing steps, Primary Antibody Enhancer was applied to slides for 10 min at RT. Following another washing, detection was achieved by incubation with the anti-mouse/rabbit UltraVision HRP-labelled polymer system (15 min) and 3-amino-9-ethylcarbacole (AEC, Dako), according to manufacturer's instructions. For negative controls, slides were incubated with negative control mouse IgG1 (Dako) at the same concentration as mentioned above. Moreover, specificity of monoclonal anti-human CX3CL1/fractalkine antibody was evaluated by an antibody preadsorption approach. For this purpose monoclonal anti-human CX3CL1/fractalkine antibody (1 : 1000, 0.5 *μ*g/mL working concentration) was mixed in Antibody Diluent with an excessive amount of recombinant human full length fractalkine (5 *μ*g/mL working concentration, rhCX3CL1/Fractalkine, R&D Systems) and incubated with gentle shaking 1 h at RT. A mixture containing solely monoclonal anti-human CX3CL1/fractalkine antibody was incubated in parallel and served as control. After incubation immunohistochemistry was performed as described above. Nuclei were stained with hemalaun and slides were mounted with Kaiser's glycerol gelatine.

### 2.3. Culture of BeWo Cells

BeWo cells were purchased from the European Collection of Cell Cultures (ECACC) and cultured in DMEM/F12 (1 : 1, Gibco), supplemented with 10% FCS, penicillin/streptomycin, amphotericin B, and L-glutamine, at 37°C in a humidified atmosphere containing 5% CO_2_ in air. Cells between passage 10 and 20 were used for* in vitro* experiments. Differentiation of BeWo cells was induced with forskolin, which was supplemented to the culture medium with a final concentration of 20 *μ*M (10 mM stock in DMSO). For experiments testing different concentrations of the metalloprotease inhibitor Batimastat (Tocris Bioscience), 1 × 10^5^ BeWo cells were seeded in 24-well culture dishes (Nunc, Thermo Fisher Scientific) and 1 mL/well of above described culture medium. For all other BeWo cell experiments, cells were seeded in 12-well culture dishes (2 × 10^5^ cells/well) and 2 mL/well culture medium. One day after seeding, cells were incubated with culture medium supplemented with or without forskolin (20 *μ*M) and Batimastat (10 *μ*M). Cells cultured in culture medium containing equal volumes of DMSO served as solvent controls. At the end of incubation conditioned culture media were collected for subsequent immunoassay measurements. Cells were washed with buffered saline and lysed with RIPA buffer (Sigma-Aldrich) including Protease Inhibitor Cocktail (Roche Diagnostics, Indianapolis, IA, USA).

### 2.4. Analysis of Cell Viability

The effect of Batimastat on viability of BeWo cells was analyzed by a methyl tetrazolium salt (MTS) based cell viability assay (CellTiter 96 AQueous One Solution Cell Proliferation Assay, Promega), according to manufacturer's protocol. In brief, 2.5 × 10^4^ BeWo cells were seeded in 100 *μ*L culture medium per well in a 96-well dish. One day after seeding, cells were incubated in culture medium supplemented with Batimastat (10 *μ*M) or solvent control DMSO (0.1%) for 48 h. After incubation, 20 *μ*L MTS solution reagent was added to each well and plates incubated for 1 h. Thereafter absorbance was recorded at 492 nm using a plate reader and absorbance values for DMSO control were set to 100%.

### 2.5. Isolation and Culture of Primary Trophoblasts

Primary trophoblasts were isolated from chorionic villi of four term placentas by enzymatic digestion and Percoll density gradient centrifugation as described previously [19]. Cells were seeded in 6-well culture dishes (3 × 10^6^/well) and 2 mL/well DMEM (Gibco) supplemented with 10% FCS and cultured in a hypoxic workstation (BioSpherix) under 8% oxygen at 37°C. One day after seeding culture medium was exchanged with DMEM/EBM (1 : 1, Gibco/Lonza) supplemented with 7.5% FCS and cells incubated for another 48 h under 8% oxygen at 37°C.

### 2.6. Placental Explant Culture

Villous tissues from human first trimester (*n* = 7, between gestational week 7 and 12) and term placentas (*n* = 3, between gestational week 38 and 40) were washed thoroughly in buffered saline and dissected into small pieces of approximately 5 mg moist mass. Placental explants were cultured in DMEM/F12 (1 : 1, Gibco) supplemented with 10% FCS, penicillin/streptomycin, amphotericin B, and L-glutamine with or without Batimastat (10 *μ*M) in a hypoxic workstation (BioSpherix) under 2.5% oxygen (first trimester explants) and 8% oxygen (term explants) for 5 days at 37°C. Placental explants cultured in culture medium containing the same volume of DMSO served as controls. After incubation, conditioned culture media were collected and placental explants homogenized in RIPA buffer with Protease Inhibitor Cocktail using a tissue homogenizer (IKA TA10 basic, Ultra-Turrax).

### 2.7. Analysis of Placental Explant Viability

Viability of placental explants was evaluated after culture by immunohistochemical staining of proliferation marker Ki67 (clone MIB-1, 1 *μ*g/mL, DAKO) and *β*hCG (clone H-298-12, 1 : 10, bioprime/biologo) as described in immunohistochemistry section. Both Batimastat treated and control explants showed proliferation of cytotrophoblasts and synthesis of *β*hCG in the syncytiotrophoblast. Moreover, effect of Batimastat treatment was analyzed and compared with DMSO control by measurement of released lactate dehydrogenase (LDH) activity in culture supernatants using LDH Cytotoxicity Detection Kit (Takara Bio Inc., eubio, Vienna; Austria) according to manufacturer's protocol. Obtained absorbance values were normalized to total protein of respective explant homogenates and DMSO control set as one.

### 2.8. Gene Expression Analysis

Total RNA from trophoblasts and placental tissues was isolated using a column based RNA isolation kit (SV Total RNA Isolation System, Promega) including an on column DNase treatment step. After quality check, total RNA was subjected to quantitative gene expression analysis using a One-Step RT-PCR Kit (Qiagen) and a predesigned expression assay for fractalkine (Hs_CX3CL1_QF_1 QuantiFast probe assay, Qiagen) according to manufacturer's instructions. In brief, 100 ng total RNA of each sample was mixed with kit components in a total reaction volume of 20 *μ*L. Samples were analyzed in triplicate in 96-well plates (Roche Diagnostics) and a Bio-Rad CXF96 Real-Time PCR System. Cycle conditions included reverse transcription for 20 min at 50°C, an initial PCR activation step for 5 min at 95°C, and subsequent 2-step cycling with denaturing for 15 s at 95°C and annealing/extension for 30 s at 60°C for a total of 40 cycles. Ct values were automatically generated by the CFX Manager 2.0 software (Bio-Rad) and relative quantification of gene expression was calculated by standard ΔΔCt method using the expression of beta-2-microglobulin (Hs_B2M_QF_2 QuantiFast probe assay, Qiagen) as reference. B2M was validated by comparison with the expression of other reference genes, ribosomal protein L30 (Hs_RPL30_QF_1 QuantiFast probe assay), hypoxanthine phosphoribosyltransferase 1 (Hs_HPRT1_QF_2 QuantiFast probe assay), and 18S rRNA (Hs_RN18S1_QF_2 QuantiFast probe assay) and showed no significant developmental or cell differentiation dependent changes.

### 2.9. Immunoblotting

Placental villous tissue was thoroughly washed with PBS and homogenized in RIPA buffer including Protease Inhibitor Cocktail using a tissue homogenizer. After determination of protein concentration according to Lowry et al., 60 *μ*g total protein was applied and separated on precast 10% Bis-Tris gels (NuPAGE, Novex; Invitrogen). 100 ng recombinant human full length fractalkine (rhCX3CL1/Fractalkine, R&D Systems) was applied as positive control. Electrophoresis was followed by semidry blotting of proteins on 0.2 *μ*m nitrocellulose membranes (Trans-Blot, Bio-Rad Laboratories). Blotting efficiency was determined by staining membranes with Ponceau S solution (Sigma Aldrich). Immunodetection was conducted with a chemiluminescent immunodetection kit (Western Breeze; Invitrogen) according to manufacturer's instructions. Monoclonal anti-human CX3CL1/fractalkine antibody (clone 81513, R&D Systems) was diluted in blocking solution 1 : 500 (1 *μ*g/mL working concentration) and applied to the membrane overnight at 4°C. For normalization membranes were incubated with monoclonal anti-beta actin antibody (1 : 20.000; clone AC-15, Abcam, Cambridge, UK). Images were acquired with FluorChem Q System (Alpha Innotech, Cell Bioscienes, Santa Clara, CA, USA) and band densities were analyzed with Alpha View SA software 3.4.0. Each tissue homogenate was analyzed in three independent immunoblot experiments. Results are presented as a ratio of relative fractalkine and beta-actin band densities, with first trimester samples set to one.

### 2.10. Fractalkine ELISA

Fractalkine was measured in cell culture supernatants and cell lysates as well as tissue homogenates using a quantitative sandwich enzyme immunoassay (Human CX3CL1/Fractalkine Quantikine ELISA, R&D Systems). Cell culture supernatants were centrifuged at 1.500 ×g and 4°C for 5 min. Cell lysates and placental explant homogenates were centrifuged at 8.000 ×g and 4°C for 10 min. After centrifugation 100 *μ*L of clear supernatants was subjected to immunoassays according to manufacturer's instruction. Complete culture medium incubated without cells and RIPA buffer served as blank for fractalkine measurement in conditioned supernatants and cell lysates, respectively. Samples were measured in duplicate and obtained fractalkine concentrations normalized to total cell protein or total tissue protein, respectively, which was determined in lysates according to Lowry method.

### 2.11. Statistical Analysis

Data were analyzed using SigmaPlot 12.5 and are presented as means ± SEM. Data were subjected to Normality Test (Shapiro-Wilk test) and Equal Variance Test. In case of normally distributed data differences between groups were tested using two-tailed* t*-test. Otherwise Mann-Whitney Rank Sum Test was applied. A *P* value of less than 0.05 was considered statistically significant.

## 3. Results

### 3.1. Spatiotemporal Fractalkine Expression in Human Placenta

**Figure 1:**
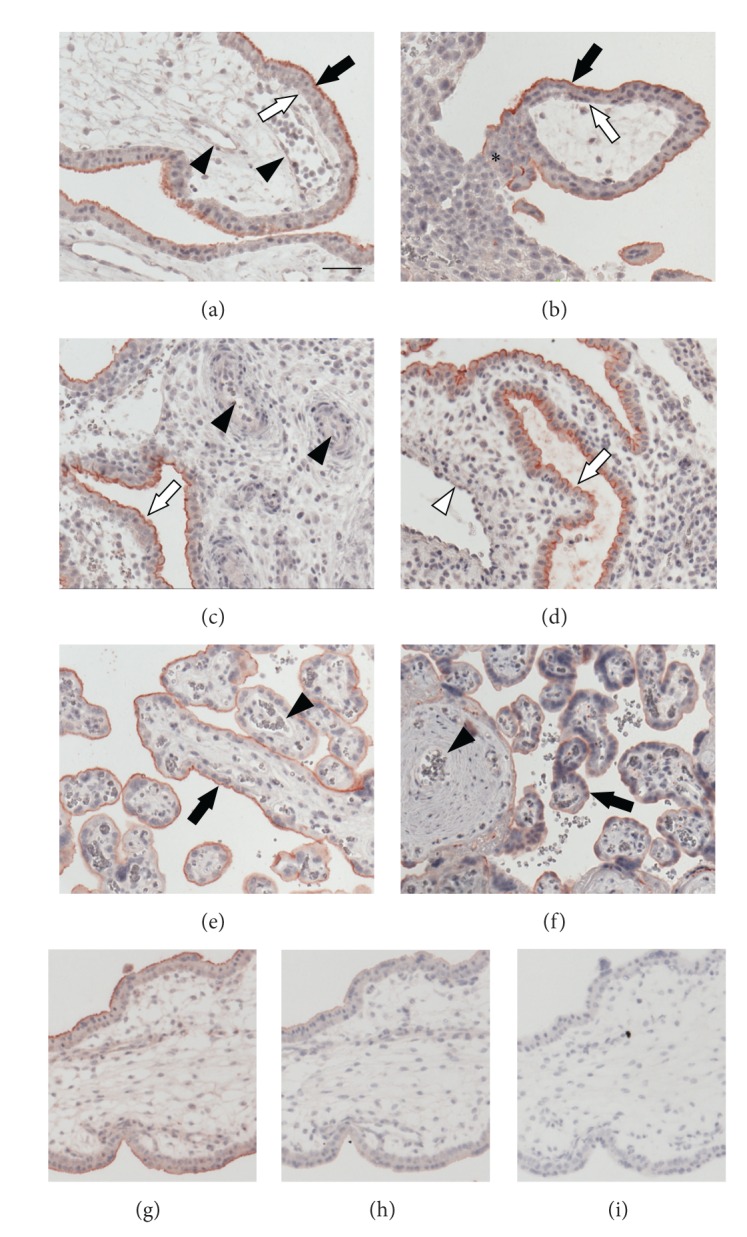
Immunohistochemical localization of fractalkine in human placenta. In first trimester placental villi fractalkine was only detected at the apical microvillous plasma membrane of the syncytiotrophoblast ((a) and (b), black arrow), but not in the fetal endothelium ((a), arrowheads), villous cytotrophoblasts ((a) and (b), open arrows), and extravillous trophoblasts in cell columns ((b), asterisk). In first trimester decidua fractalkine was detected at the apical plasma membrane of uterine glandular epithelial cells ((c) and (d), open arrows), whereas spiral arteries ((c), arrowheads) and veins ((d), open arrowheads) did not show any staining. In term placenta fractalkine was detected at the apical plasma membrane of the syncytiotrophoblast ((e) and (f), black arrows). The fetal vascular endothelium of all villous types including terminal villi ((e), arrowheads) and stem villi ((f), arrowhead) did not express fractalkine. Specificity of the antibody was evaluated on serial first trimester placenta sections using an antibody preadsorption approach and negative control mouse IgG, respectively. While incubation with the antibody alone confirmed the above described staining pattern (g), preadsorption of the antibody with recombinant fractalkine almost completely abolished staining (h). Incubation with negative control mouse IgG revealed no staining (i). Scale bar represents 50 *μ*m.

Immunohistochemical staining of human first trimester placental sections localized fractalkine at the apical microvillous plasma membrane of the syncytiotrophoblast (Figure 1(a)). The fetal endothelium, villous cytotrophoblasts, and extravillous trophoblasts in cell columns did not express fractalkine (Figures 1(a) and 1(b)). In first trimester decidua fractalkine was detected at the apical plasma membrane of uterine glandular epithelial cells (Figure 1(c)). Neither spiral arteries nor uterine veins showed endothelial staining (Figures 1(c) and 1(d)). In human term placenta fractalkine was detected at the apical plasma membrane of the syncytiotrophoblast (Figure 1(e)). No staining was observed in the fetal vascular endothelium of terminal villi and stem villi (Figures 1(e) and 1(f)).

**Figure 2:**
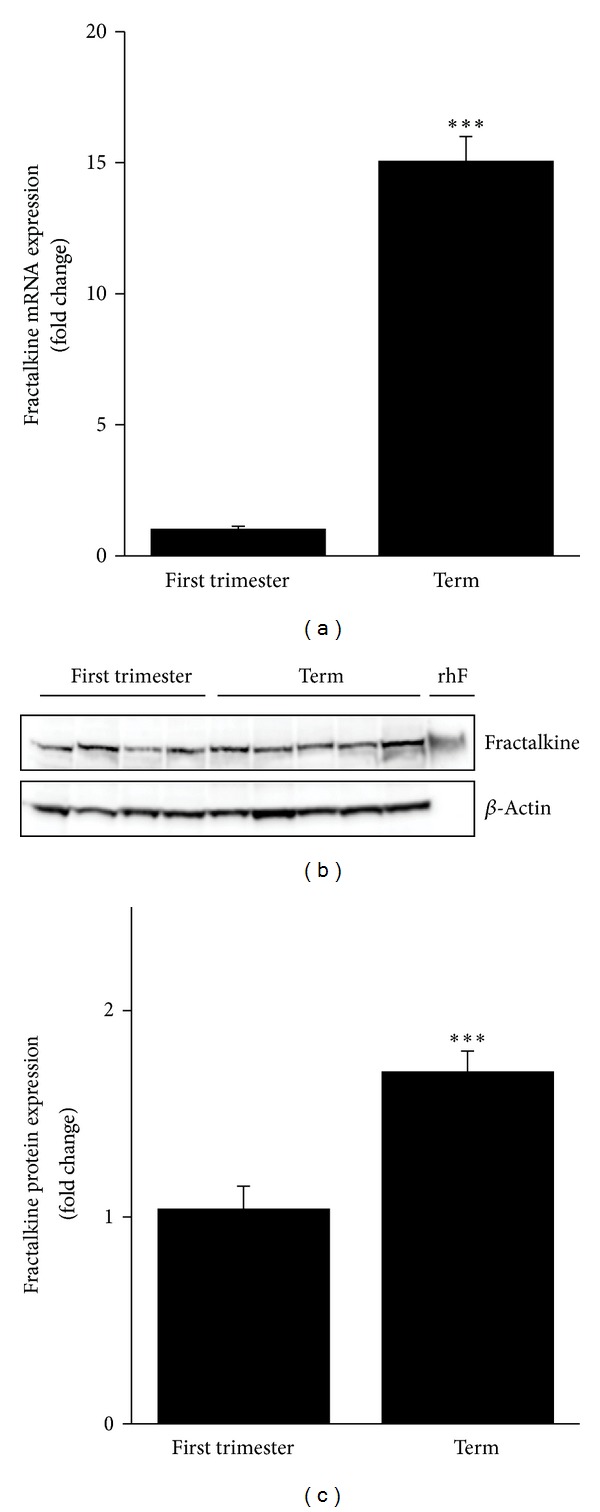
Analysis of fractalkine expression in human first trimester and term placenta tissue. Quantitative real-time RT-PCR analysis showed a 15.1-fold (±0.9) increase of fractalkine mRNA expression in term (*n* = 8) compared to first trimester (*n* = 9) placental tissues (a). Immunoblot analysis of first trimester and term placenta tissue homogenates detected a protein band, corresponding with recombinant 90 kDa human full length fractalkine (rhF), which was applied as positive control. Shown is one representative immunoblot (b). Semiquantitative band analysis revealed a 1.7-fold (±0.1) increase in fractalkine protein expression at term (*n* = 7) compared to first trimester (*n* = 8) (c). Values are given as mean ± SEM. ***P ≤ 0.001.

To get an idea of putative changes of placental fractalkine expression over gestation, placental tissues were analyzed at first trimester and term. Quantitative gene expression analysis revealed a 15.1-fold (±0.9) increase in placental fractalkine mRNA expression at term, when compared to first trimester (Figure 2(a)). On protein level, placental fractalkine was detected by immunoblotting of first trimester and term placenta tissue homogenates and corresponded with recombinant 90 kDa full length fractalkine, which served as positive control (Figure 2(b)). In contrast to quantitative gene expression analysis, semiquantitative band densitometry of immunoblots showed only a 1.7-fold (±0.1) increase of placental fractalkine at term, when compared to first trimester (Figure 2(c)).

### 3.2. Fractalkine Expression in the Trophoblast Cell Line BeWo and Primary Term Trophoblasts

**Figure 3:**
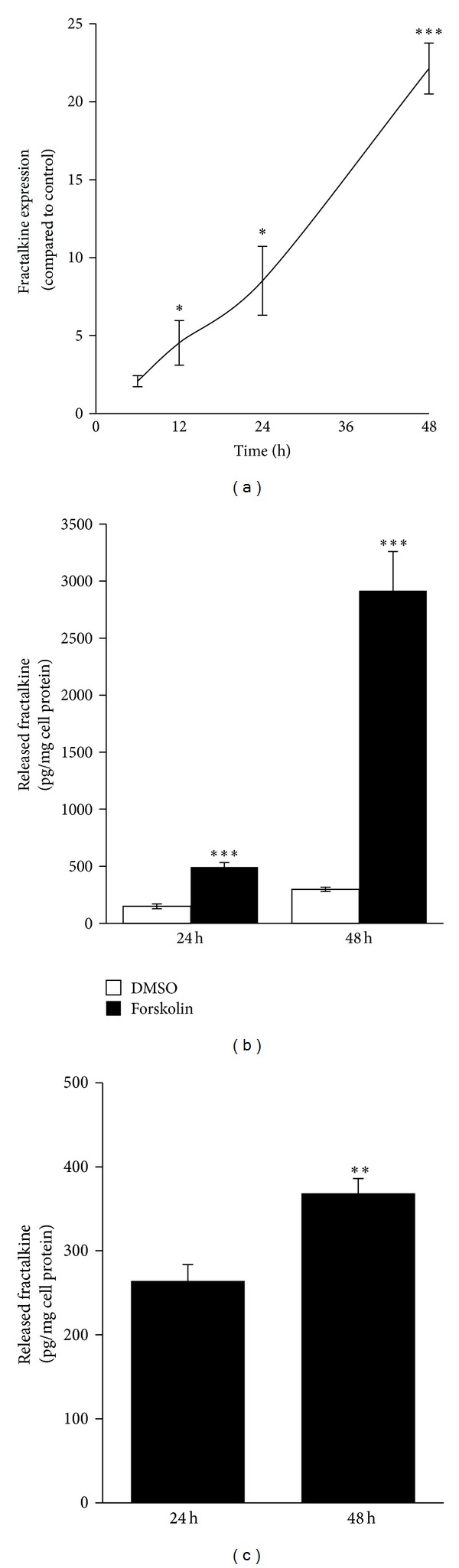
Analysis of fractalkine expression and release in the trophoblast cell line BeWo and primary term trophoblasts. Incubation with forskolin (20 *μ*M) increased fractalkine mRNA expression in BeWo cells in a time dependent manner and showed a 22.1-fold (±1.6) upregulation compared to control (DMSO, 0.2%) after 48 h (a). Forskolin treatment of BeWo cells induced a 9.8-fold increase in secretion of soluble fractalkine compared to control after 48 h (b). In primary term trophoblasts secretion of soluble fractalkine increased between 24 h and 48 h incubation by 39.5% (c). Data in (a) and (b) are presented as mean ± SEM from three independent experiments performed in triplicate. Data in (c) are presented as mean ± SEM from four different trophoblast preparations. *P ≤ 0.05, **P ≤ 0.01, ***P ≤ 0.001.

Immunohistochemistry suggested the syncytiotrophoblast to be the main source of placental fractalkine expression. In order to substantiate this finding the trophoblast cell line BeWo, a well accepted model for the villous trophoblast population, showing secretion of pregnancy-specific hormones as well as good syncytialization, that is, formation of trophoblast syncytia* in vitro* [20], was tested for its capacity to express and release the chemokine. While basal expression of fractalkine mRNA was low but detectable in untreated BeWo cells, incubation with forskolin, a reagent known to induce BeWo cell differentiation and syncytialization [21], led to a time dependent increase over time with a 22.1-fold (±1.6) upregulation compared to vehicle control after 48 h (Figure 3(a)). Forskolin induced fractalkine expression in BeWo cells was accompanied by a 9.8-fold increase in the release of soluble fractalkine, which accounted for 2.91 (±0.34) ng/mg cell protein after 48 h incubation (Figure 3(b)).

Since trophoblast cell lines may differ in some aspects when compared to their primary counterpart [22], primary trophoblasts shown to spontaneously form syncytia* in vitro* [23] were isolated from term placenta and tested for their capacity to release soluble fractalkine. Analysis of supernatants from primary term trophoblasts showed continuous release of soluble fractalkine, which increased by 39.5% between 24 h and 48 h of incubation (Figure 3(c)).

### 3.3. Inhibition of Placental Fractalkine Release by the Metalloprotease Inhibitor Batimastat

**Figure 4:**
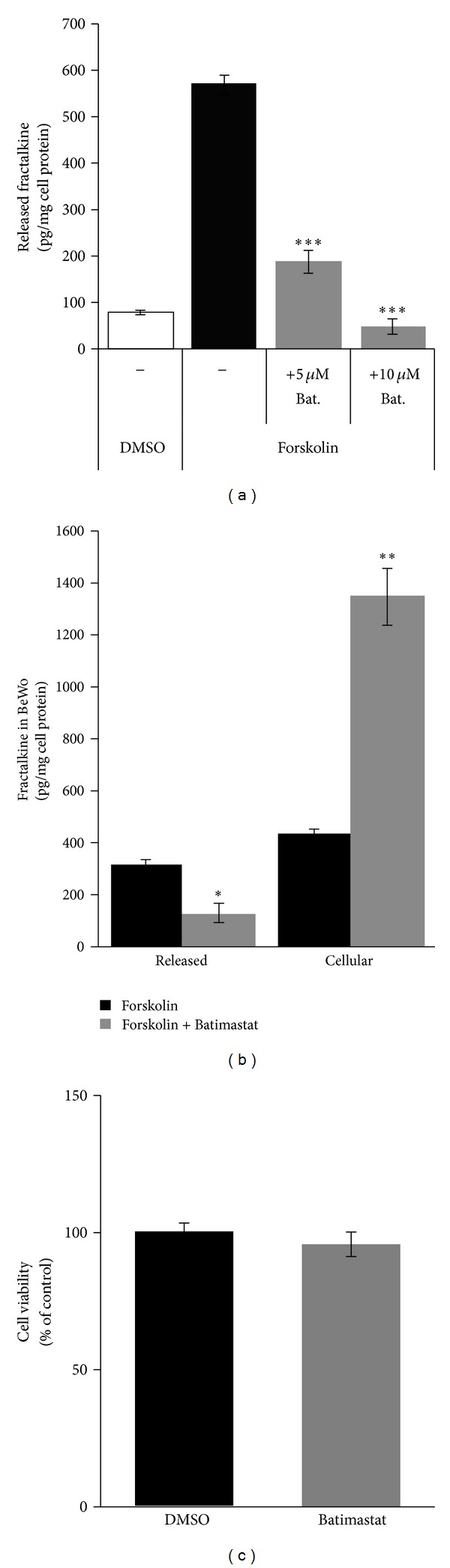
Effect of Batimastat on the release of soluble fractalkine in the trophoblast cell line BeWo. Increasing concentrations of the metalloprotease inhibitor Batimastat (5 *μ*M and 10 *μ*M, resp.) inhibited forskolin (20 *μ*M) induced upregulation of fractalkine release in BeWo cells after 48 h (a). Inhibition of fractalkine release by Batimastat (10 *μ*M) was accompanied with an increase in cell bound fractalkine in BeWo cells after 48 h (b). Batimastat (10 *μ*M) slightly but not significantly decreased viability of BeWo cells compared to control (*P* = 0.456) (c). Data are presented as mean ± SEM from three independent experiments performed in triplicate. *P ≤ 0.05, **P ≤ 0.01, ***P ≤ 0.001.

Data from trophoblast culture provided strong evidence that fractalkine is not only expressed but also released from villous trophoblast. Since data on placental fractalkine release have virtually not been described so far, it was tempting to test if the previously described mechanism of metalloprotease mediated shedding of the transmembrane form also applies for human placenta, that is, human trophoblast. For this purpose the release of soluble fractalkine was first analyzed in forskolin treated BeWo cells in presence and absence of the metalloprotease inhibitor Batimastat, which has previously been shown to effectively block fractalkine shedding in other cell types such as smooth muscle cells and hepatic stellate cells [24, 25]. Incubation with 5 *μ*M and 10 *μ*M Batimastat decreased the release of soluble fractalkine by 67.1% and 91.6%, respectively, compared to cells treated without the inhibitor after 48 h (Figure 4(a)). Analyses of cell lysates from forskolin stimulated BeWo cells incubated with or without Batimastat revealed that inhibition of soluble fractalkine release was accompanied by a 3.1-fold increase of cell associated fractalkine compared to control after 48 h (Figure 4(b)). To ensure that observed effects were not due to changes in cell viability, Batimastat treated and control cells were analyzed using a MTS based viability assay. Accordingly, Batimastat treatment slightly but not significantly decreased the relative number of viable BeWo cells in proliferation by 4.3% compared to control after 48 h (Figure 4(c)).

**Figure 5:**
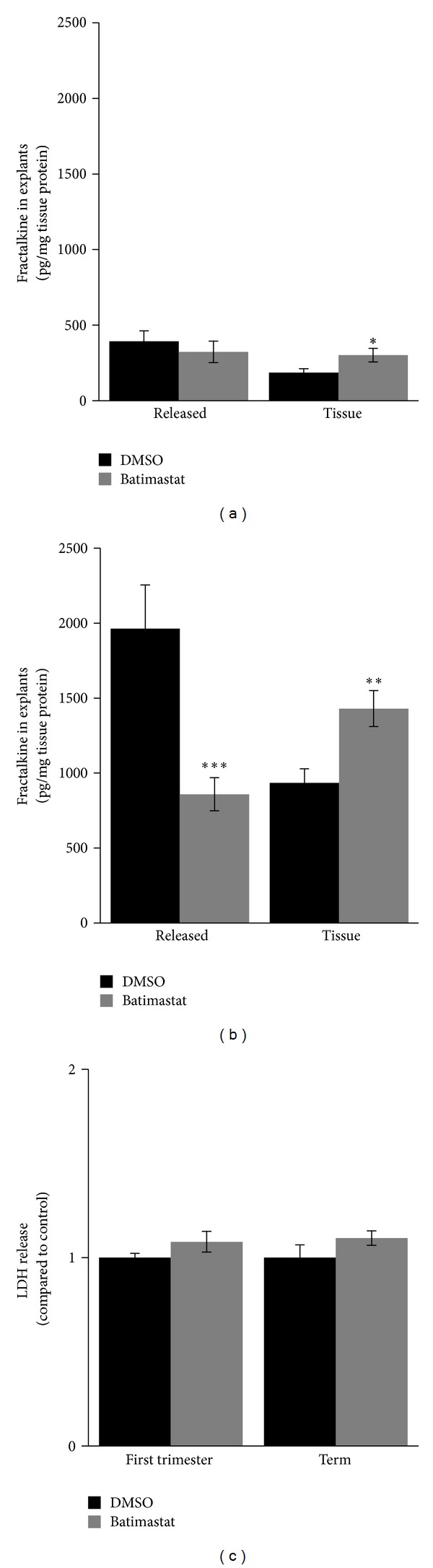
Effect of Batimastat on the release of soluble fractalkine in placental explants. In first trimester placental explants Batimastat (10 *μ*M) slightly but not significantly decreased the release of soluble fractalkine, whereas at the same time tissue associated fractalkine increased, compared to control (DMSO, 0.1%) after 5-day culture (a). Incubation of term placental explants with Batimastat (10 *μ*M) led to a considerable decline in fractalkine release and was accompanied with an increase of the tissue associated fractalkine fraction after 5-day culture (b). Analysis of culture media from first trimester and term placental explants showed slight but not significant increase of released LDH activity in presence of Batimastat (10 *μ*M) compared to control after 5-day culture (c). Data in (a) are presented as mean ± SEM from seven different explant experiments performed in triplicate. Data in (b) and (c) are presented as mean ± SEM from three different explant experiments performed in triplicate. *P ≤ 0.05, **P ≤ 0.01, ***P ≤ 0.001.

The situation observed in BeWo cells was at least in part reflected in explant culture of human first trimester and term placenta. In first trimester placental explants, Batimastat decreased the release of soluble fractalkine by 17.8% after 5-day culture, which did not reach statistical significance. However, analyses of respective tissue homogenates revealed a significant 1.6-fold increase of tissue associated fractalkine in explants incubated with Batimastat, when compared to controls after 5 days (Figure 5(a)). In explants from human term placenta, incubation with Batimastat showed a considerable decline of soluble fractalkine release by 56.3%, while at the same time the fraction of tissue associated fractalkine increased 1.5-fold when compared to controls (Figure 5(b)). Comparison of controls from first trimester and term explant experiments revealed a 5.0-fold increase in tissue associated as well as released fractalkine towards term. However, the ratio of released versus tissue associated fractalkine remained constant and was 2.1 in both first trimester and term placental explants, suggesting constitutive shedding of placental fractalkine.

In order to determine any cytotoxic effects of Batimastat on placental explants, the release of LDH into the culture medium was analyzed after culture and showed a slight but nonsignificant increase by 8.5% and 10.4% after Batimastat treatment of first trimester and term explants, respectively, when compared to controls (Figure 5(c)).

## 4. Discussion

The concept of the dual nature of fractalkine, acting both as soluble chemoattractive factor and transmembrane adhesion molecule, can be well applied for fractalkine expressed in human placenta. Data from placental explant and trophoblast culture provide strong evidence that placental fractalkine is constitutively released from the syncytiotrophoblast into the maternal circulation via metalloprotease dependent shedding. When speculating about a putative role of shed placental fractalkine in the fetal-maternal cross-talk, important aspects of placental development should be considered. During early pregnancy perfusion of the intervillous space with maternal blood is not yet fully established, and thus shed placental fractalkine may not act locally on maternal CX3CR1 expressing cells, but rather in an endocrine way. In doing so, placenta derived fractalkine may contribute to the low grade systemic inflammatory responses described to occur in third trimester of pregnancy [26–28]. This assumption is in good agreement with increasing expression and release of placental fractalkine towards term.

Mild inflammatory responses were suggested to contribute to maternal metabolic changes, resulting in insulin resistance and hyperlipidaemia, which accommodate increased energy demands of the growing fetus. Contribution of placental fractalkine to the maternal pool of soluble fractalkine during gestation is hard to estimate and should include the fact that the entire surface of placental villi at term with approximately 12–15 m^2^ represents only a very small area compared to approximately 4000–7000 m^2^ endothelium of maternal blood vessels [1, 29]. Expression analysis of other proinflammatory cytokines, such as TNF-*α*, IL-6, IL-1*α*, and IL-1*β*, showed no difference between preeclamptic and normal placental explants, suggesting a rather marginal contribution of placenta derived cytokines to systemic inflammation [30].

Based on placental explant experiments, the ratio between shed to membrane-bound fractalkine seems to remain constant from first trimester until term but may be influenced by parameters such as gene expression, half-life of both variants, and metalloprotease dependent shedding. The phenomenon of increased membrane-bound fractalkine in cells treated with Batimastat was explained as an accumulation of the membrane-bound form as a consequence of impaired shedding activity on the cell surface [24, 25]. Since shedding is mediated by a disintegrin and metalloprotease (ADAM)10 and ADAM17 [8, 9], which both can be detected in the syncytiotrophoblast, it is tempting to speculate about an aberrant activity of these metalloproteases and its consequence on the release of placental fractalkine in pathological pregnancies. Interestingly, expression of both metalloproteases has been shown to be increased in placentas from pregnancies complicated by preeclampsia [31, 32], suggesting increased shedding and release of placental fractalkine. This assumption is in line with a recent case-control study, showing elevated plasma concentrations of soluble fractalkine in women with preeclampsia [33]. However, whether or not preeclampsia is accompanied with increased release of placental fractalkine remains open and requires further in-depth studies.

Detection of fractalkine expression in uterine glandular epithelial cells is in good agreement with previous studies showing fractalkine in apical regions of the glandular epithelium of actively secreting glands of nonpregnant endometrium as well as in early decidua [34]. When speculating about a physiological function of fractalkine release by uterine glands it should be considered that early implantation processes, with the enlarging syncytiotrophoblast invading not only uterine capillaries but also uterine glands, give rise to connections between the latter and the intervillous space. This situation can be observed from approximately day 17 after conception throughout the first trimester, suggesting delivery of glandular secretion products, including nutrients, growth factors, and immunomodulatory cytokines into the intervillous space during first and early second trimester [35, 36]. Recently, replacement of glandular epithelial cells by so-called endoglandular trophoblasts has been suggested as additional mechanism for opening and connection of the uterine glands towards the intervillous space [37]. Thus, uterine glands together with the growing syncytiotrophoblast may contribute to a continuous release of fetal fractalkine into the intervillous space, that is, maternal plasma. At this stage of pregnancy autocrine signalling by placental fractalkine may not completely be excluded, since a study by Hannan et al. showed weak CX3CR1 staining in the villous trophoblast layer of human first trimester placenta [16]. However, with ongoing pregnancy autocrine effects of placenta derived fractalkine may be neglected, as CX3CR1 can only be detected in the fetal endothelium but not the villous trophoblast compartment at term [18, 38].

While placental fractalkine may contribute as soluble factor to low grade systemic inflammatory responses in the mother, its role as membrane-bound chemokine located on the surface of placental villi is rather unclear. Adhesion of maternal leukocytes to the syncytiotrophoblast may be considered—if at all—as very rare event in normal pregnancy. However, mechanisms preventing CX3CR1 expressing maternal leukocytes from binding to the syncytiotrophoblast remain speculative. Specific glycans, like sialyl Lewis X and Lewis a on glycosylated proteins, such as hCG, have recently been suggested to play a role in prevention of maternal leukocyte adhesion to trophoblast [39]. Nevertheless, under pathological conditions membrane-bound fractalkine could facilitate adhesion and transmigration of maternal leukocytes through the villous trophoblast layer giving rise to accumulation of maternal immune cells within inflamed villi, as has been described for infectious villitis and villitis of unknown etiology [40–42].

## 5. Conclusion

The human placenta is a source for the chemokine fractalkine, which is expressed in the syncytiotrophoblast and released into the maternal circulation by metalloprotease dependent shedding. Increased expression and release of placental fractalkine may contribute to low grade systemic inflammatory responses observed in third trimester of normal pregnancy. Aberrant placental metalloprotease activity may not only affect the release of placenta derived fractalkine but may at the same time affect the abundance of the membrane-bound form of the chemokine.
